# Lessons from genome-wide studies: an integrated definition of the coactivator function of histone acetyl transferases

**DOI:** 10.1186/1756-8935-3-18

**Published:** 2010-10-20

**Authors:** Krishanpal Anamika, Arnaud R Krebs, Julie Thompson, Olivier Poch, Didier Devys, Làszlò Tora

**Affiliations:** 1 Department of Functional Genomics and Cancer, Institut de Génétique et de Biologie Moléculaire et Cellulaire (IGBMC), CNRS UMR 7104, INSERM U 964, Université de Strasbourg, 1 Rue Laurent Fries, 67404 Illkirch Cedex, France; 2 Department of Integrated Structural Biology, Institut de Génétique et de Biologie Moléculaire et Cellulaire (IGBMC), CNRS UMR 7104, INSERM U 964, Université de Strasbourg, 1 Rue Laurent Fries, 67404 Illkirch Cedex, France

## Abstract

Histone acetylation is one of the key regulatory mechanisms controlling transcriptional activity in eukaryotic cells. In higher eukaryotes, a number of nuclear histone acetyltransferase (HAT) enzymes have been identified, most of which are part of a large multisubunit complex. This diversity, combined with the large number of potentially acetylable lysines on histones, suggested the existence of a specific regulatory mechanism based on the substrate specificity of HATs. Over the past decade, intensive characterisations of the HAT complexes have been carried out. However, the precise mode of action of HATs, and particularly the functional differences amongst these complexes, remains elusive. Here we review current insights into the functional role of HATs, focusing on the specificity of their action. Studies based on biochemical as well as genetic approaches suggested that HATs exert a high degree of specificity in their acetylation spectra and in the cellular processes they regulate. However, a different view emerged recently from genomic approaches that provided genome-wide maps of HAT recruitments. The careful analysis of genomic data suggests that all HAT complexes would be simultaneously recruited to a similar set of loci in the genome, arguing for a low specificity in their function. In this review, we discuss the significance of these apparent contradictions and suggest a new model that integrates biochemical, genetic and genome-wide data to better describe the functional specificity of HAT complexes.

## Introduction

Histone post-translational modifications have shown to be key regulators among transcription regulation mechanisms [1,2]. Histone acetylation is known to play an important role in the regulation of transcriptional activity in eukaryotic cells [3] by affecting higher-order folding of chromatin fibres, loosening of the contacts between the DNA and the nucleosomes and/or histone-nonhistone protein interactions [4-8]. Histone acetylation on various target lysines is in general positively associated with gene expression. Thus, HATs (also called lysine acetyl transferases or KATs) are thought to increase the decompaction of chromatin, which in turn may increase the accessibility of factors that promote transcription [8-11]. In higher eukaryotes, two enzymatic families (GNAT and MYST), each containing a dozen histone acetyltransferase (HAT) enzymes, have been identified and have often been shown to be subunits of larger transcriptional coactivator complexes.

Over the past decade, two approaches were mainly used to better understand the functional specificity of HATs. First, *in vitro *acetylation assays were carried out to investigate the substrate specificity of distinct HATs. These analyses showed that HATs exert a certain degree of specificity for particular lysine residues on different histone tails. Second, *in vivo *gene inactivation studies allowed testing the HAT specificity by observing phenotypical effects caused by ablation of a particular HAT. Interestingly, most of these studies argued for a high degree of specificity in the developmental or gene expression phenotypes. More recently, availability of new high-throughput technologies such as chromatin immunoprecipitation sequencing (ChIP-seq) allowed the investigation of the recruitment of HATs and the deposition of acetylation marks at a genome-wide scale [12,13]. Contrary to the biochemical and genetic evidence, when carefully analysed, the genome-wide data suggest a low specificity in the recruitment and activity of HATs.

Here we comparatively review the conclusions of the above-mentioned three different approaches. Additionally, we discuss the significance of the conclusions made from each approach and try to reconcile new genome-wide evidence with existing knowledge in the form of a new model for the mode of action of HATs in transcriptional activation.

### Biochemistry: Each HAT has a specific histone acetylation spectrum modulated by its macromolecular complex

**Figure 1:**
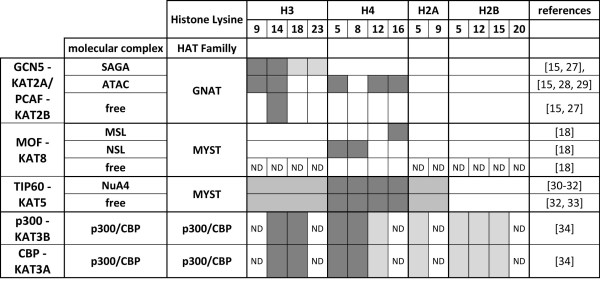
**Substrate specificity of histone acetyltransferases (HATs) *in vitro***. Review of the *in vitro *substrate specificity described in mammalian systems for the studied five HAT enzymes, either as a free protein or within their respective macromolecular complexes. Dark grey boxes represent lysine residues highly acetylated, light grey boxes represent residues where weak acetylation activity has been observed, and white boxes represent residues where no acetylation is detected. ND represents residues that have not been tested for acetylation. References [15,18,27-34] cited in figure.

The two aspects that were intensively investigated immediately after the discovery of HATs were (1) the identification of the protein complexes in which the individual HAT enzyme is incorporated and (2) the determination of the acetylation spectrum associated with each HAT individually and then in the corresponding complexes (both reviewed in Figure 1 for mammalian cells). Several conclusions emerged from these studies. First, it was shown that most of the HAT enzymes are part of large multisubunit complexes. For example, Tip60 (Tat-interactive protein 60 kD; also called KAT5) is contained in the 18 subunits of the NuA4 complex [14], whereas GCN5 (general control of amino-acid synthesis 5; also called KAT2A) or PCAF (p300/CBP-associated protein; also called KAT2B) are members of the 19 subunits of the SAGA complex and the 10 subunits of the ATAC complex [15]. Second, several studies described how the association of the HAT enzymes with their partners in the corresponding complexes can modify the specificity of the HATs on histones (i.e., *Drosophila *GCN5 was shown to have different targeting specificity in ATAC or SAGA complexes [16]). Third, each HAT acetylates a defined spectrum of target lysines, and these acetylation sites only partially overlap between individual HAT complexes (see Figure 1 for review in mammalian systems). Taken together, these observations suggest a high specificity of each HAT when incorporated in its corresponding macromolecular complex, suggesting that this integration is required for the full and specific activity *in vivo*. Moreover, these studies suggest that each HAT complex should create a specific signature on its target loci because a distinguishable acetylation pattern is observed for each complex tested *in vitro *[11,17].

### Genetics: HATs exert a high degree of functional specificity

**Figure 2:**
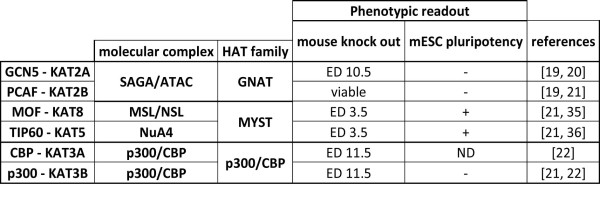
**Phenotypes observed in gene disruption studies of HATs**. Phenotypes associated with HAT genetic knockout (KO) or RNAi targeting in mouse development and embryonic stem cell (ESC) pluripotency, respectively. For mouse KO, the day of embryonic death (ED) for homozygous animals is presented as a phenotype readout parameter. For the effect on mouse embryonic stem cell (mESC) pluripotency, any observation (e.g., flattening of the cells, differentiation) was simplified as a positive phenotype (+). ND represents HATs for which the parameter was not determined. References [19-22,35,36] cited in figure.

Another aspect that was systematically addressed to better understand the function of HATs is the phenotypical analysis of the effect of distinct ablation of HATs *in vivo *(reviewed in Figure 2). Genetic knockout (KO) studies of HATs during mouse development reveal a significant variability in the phenotypes of HAT ablations. For example, *Tip60*^-/- ^embryos do not develop beyond blastocyste stage (i.e., they die at embryonic day (ED) 3.5) [18], while *Gcn5*^-/- ^embryos die at around ED 10.5 [19]. Moreover, the maintenance of embryonic stem cell (ESC) pluripotence can be used as a second phenotypical readout. Using RNAi knockdown of Tip60, this enzyme was shown to be required for ESC pluripotency, while GCN5 or PCAF seems to be dispensable (Figure 2) [20,21]. Together, these results suggest that each HAT has key roles at different stages of development that seem to be independent of and distinct from other HATs.

A remarkable feature emerging from sequence alignment of vertebrate HATs is the presence of highly related paralogs [i.e., CBP (CREB-binding protein, KAT3A)/p300 (KAT3B) or Gcn5/PCAF], whereas for most of the nuclear HATs only a single gene is present in nonvertebrate species. This observation raised the question of the functional importance of closely related paralogs in vertebrates. Answers were partially obtained by crossing mice carrying individual homozygous or heterozygous KOs of the given paralogs and comparing double KOs to that of the single KOs. For example, while the single CBP or p300 heterozygous mutants exert a milder phenotype than the homozygous mutants, the mice heterozygous for both HATs show phenotypes similar to the homozygous depletion of one or the other paralog [22]. This result suggests that there is a functional redundancy between these paralogs and that in certain cases the dose of expression of the two paralogs is crucial for the proper development of the animals.

Taken together, these studies suggest that, amongst HATs, a high degree of functional specificity exists, with the exception of closely related paralogs that rather reflect the importance of gene expression dosage than functional specificity.

### Genome-wide mapping: HATs are co-occurring at high frequency, creating hyperacetylated environment

In the past few years, our understanding of genome regulation has tremendously progressed due to the introduction of ChIP combined with microarray (ChIP-chip) or with high-throughput sequencing (ChIP-Seq). These experiments have allowed us to analyse the presence of a particular genomic feature at the genome-wide scale. Recently, several systematic ChIP-seq studies have been published, providing genome-wide mapping data for HATs and acetylation marks in resting human T cells [12,13].

**Figure 3:**
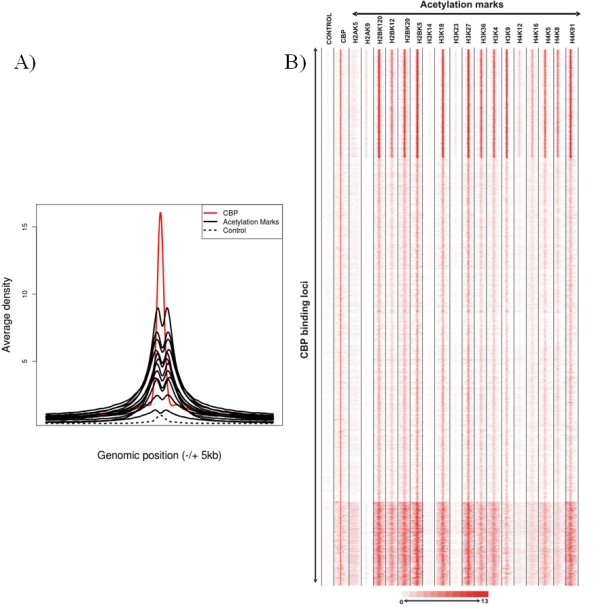
**Similar genome-wide binding patterns of 15 HAT marks at binding sites of HATs**. Raw chromatin immunoprecipitation sequencing (ChIP-seq) data were extracted from [12,13]. **(a) **Co-occurrence of 15 acetylation marks on CBP binding sites: average binding densities of 15 HAT marks (marked in black), control (immunoglobulin G (IgG)) (marked in dashed black) and CBP (marked in red) surrounding ± 5-kb region of a collection of 10,360 CBP binding sites. From the raw data sets, enrichment clusters representing CBP binding sites were determined. Around each CBP binding site, four hundred 25-bp bins were created, and densities were collected for each bin for the 15 acetylation tracks. The mean was calculated for each bin and used to represent average acetylation densities around the CBP binding sites. **(b) **Co-occurrence of 18 acetylation marks on all the genome-wide CBP binding sites: binding densities of regions (± 5) surrounding the 10,360 binding sites of CBP. Densities are shown for control (IgG), CBP and 18 HAT marks (as indicated). In the heat map, each line represents a genomic location of a binding site with its surrounding ± 5-kb region. CBP binding sites were used as references to collect ChIP-seq tag densities over a 10-kb (± 5 kb) window. This matrix was subjected to *k*-means clustering. The heat map representing the clustered density matrix is displayed. In **(a) **and **(b)**, similar results were obtained with the other four other HATs (data not shown).

**Figure 4:**
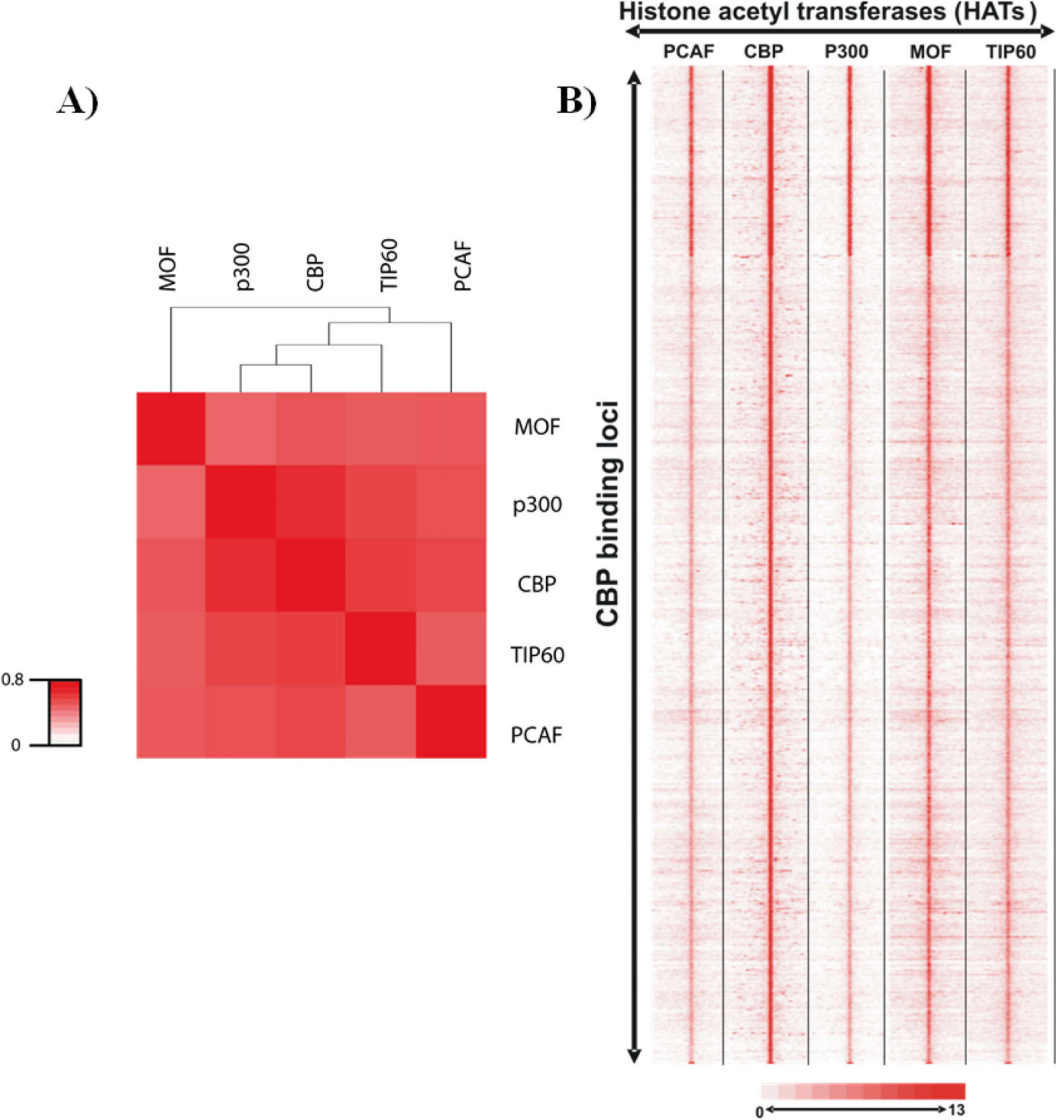
**HATs are corecruited at high frequency on their binding loci**. Raw ChIP-seq data were extracted from [12,13]. **(a) **Heat map showing colocalization frequency of all the five HATs, namely, MOF, p300, CBP, TIP60 and PCAF. Colors in the heat map reflect the colocalization frequency (Pearson correlation coefficient) of each pair of HAT (red means more frequently colocalized) over a 5-kb region surrounding the complete set of HAT binding sites. HATs were clustered along both axes based on the similarity in their colocalization with other HATs. **(b) **Co-occurrence of five HATs on CBP binding sites: Binding densities of PCAF, p300, MOF and TIP60 were clustered according to 10,360 CBP binding sites. In the clustering, each line represents a genomic location of a binding site with its surrounding ± 5-kb region. HAT binding sites were used as a reference to collect ChIP-seq tag densities over a 10-kb (± 5 kb) window in each HAT density map. This matrix was subjected to *k*-means clustering. The heat map representing the clustered density matrix is displayed.

This resource has given us the opportunity to analyse the specificity of genome-wide HAT recruitments. We have first extracted genome-wide distributions of five HATs [namely, p300, CBP, MOF (males absent on the first; KAT8), PCAF, and Tip60) and 18 acetylation marks and then systematically analysed the presence of each of the 18 acetylation marks at the binding sites of the five distinct HATs. Interestingly, this analysis revealed that, when comparing the genome-wide binding sites of a given HAT with that of the 18 different histone acetylation marks, no specific or unique acetylation pattern for any of the five HATs could be identified (Figures 3a and 3b; shown for CBP). In each of these "HAT-acetylation mark" comparisons, variations in acetylation levels between different categories of loci can be observed, but with the same patterns for all the residues tested (except for histone H2AK5ac, H3K14ac and H3K23ac for which the antibodies used in the ChIP may have been too weak for the analysis). The most likely hypothesis to explain these results is that HATs are co-occurring at these sites. To further analyse this possibility, the binding specificity of a given HAT versus the four other HATs has been tested. Interestingly, this analysis revealed that each genomic locus bound by a given HAT is also bound to some extent by the four other HATs (Figures 4a and 4b). It appears that the binding of each HAT correlates with the binding of all the other HATs tested (Figure 4a; Pearson correlation > 0.6).

The detected co-occurrence of HATs and the consequent broad acetylation patterns observed can suggest two different scenarios. First, since in ChIP cell populations rather than a single cell are analysed, we cannot exclude that the recruited HATs could occur in a stochastic manner. This would imply that in a given cell, one HAT is recruited to a particular locus, while in another cell, a distinct HAT is recruited instead to the same locus. In this scenario, the observed lack of histone acetylation specificity would come from the lack of specificity in the mechanisms that recruit HATs to particular loci. In this case, the acetylation specificity would not be an important feature for regulation, but only the acetylation *per se *would be requested for proper activation.

In the second scenario, HATs would work collaboratively. In all cells, all the studied HATs would be permanently recruited and released at every bound loci in a dynamic fashion. This scenario implies also that many transcriptional coactivators can be dynamically recruited all the time to a set of regulated loci to modify (acetylate) the given chromatin environment. Such a mechanism for coactivator action has already been suggested by Hager *et al. *[23]. Thus, gene regulation would mainly rely on the local abundance of the different coactivators rather than on the recruitment of a specific coactivator and its corresponding specific pattern of histone modifications. In this case, there would be a collaborative effect of all the HATs on the deposition of histone acetylation marks at the regulated set of genes.

### An integrated model depicting mode of action of HATs

**Figure 5:**
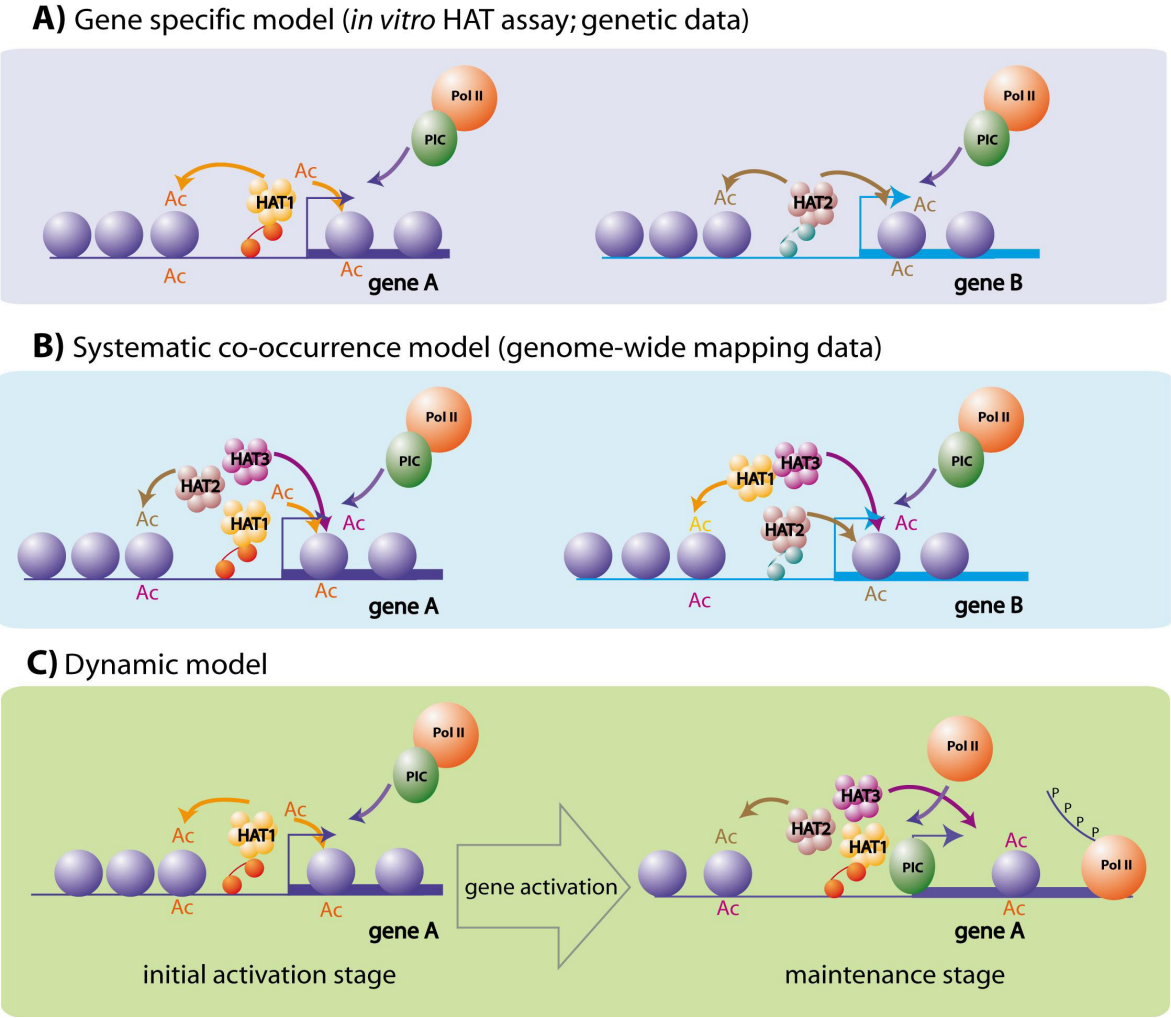
**Schematics describing the possible modes of action of HATs**. **(a) **Gene specificity model. This model can be proposed on the basis of the conclusions from biochemical and genetic studies. Each HAT complex is recruited by a particular DNA binding transcriptional activator to a defined set of genes, allowing their activation. Thus HATs seem to exert a high functional specificity. **(b) **Systematic co-occurrence model. This model arises from conclusions of genome-wide mapping studies. HATs would be corecruited to all the transcriptionally active loci, creating a hyperacetylated environment that would favor the activation of the corresponding genes. **(c) **Dynamic model. Model proposed to reconcile the observations of the genome-wide mapping of HATs with previous biochemical and genetic observations. HATs play a dual role in the gene activation process. In the first phase, a specific HAT recruited by a specific activator allows the initiation of the activation process. Later other HATs can bind the activated loci in a less-specific manner, thus maintaining a nonspecific hyperacetylated environment.

Taken together, the evidence emerging from genomics, biochemical and genetic analyses of HAT action result in apparently contradictory conclusions. While both the biochemistry and the genetic data argue for a rather high degree of specificity in the functional spectrum of HATs (Figure 5a), the current genome-wide evidence suggests that HATs are in general redundantly recruited to the same set of loci (Figure 5b), suggesting low specificity in the action of HATs. To reconcile the different observations, we first confront each line of evidence and then propose a new model that may better explain the *in vivo *action of HAT complexes (Figure 5c).

The genome-wide results do not directly contradict the biochemical evidence, since the broad *in vivo *co-occurrence of HAT enzymes at many genomic loci would explain the observation of multilysine hyperacetylated loci *in vivo*. However, this model is challenged by the conclusions of the genetic studies. It is clear from the KO studies that different HATs cannot compensate for each other's function. Moreover, if each process would require all the HATs, one would expect that all HAT KOs would result in the same phenotype, since all HAT-dependent processes should be affected. Thus a model assuming a complete overlap in HAT function can be excluded, and new hypotheses have to be raised to integrate the new lines of genome-wide evidence.

The time is a variable that was not introduced in the previous formulation of hypotheses. The analysed ChIP-seq data were all produced at a time *t *that reflects a single stage of the transcription activation process. Since the analysed genome-wide data were generated in resting somatic cells (CD4+ T cells) that are not challenged, one can assume that they exert only "routine" gene expression programs compared to the major transcriptional changes happening during differentiation of a tissue or an organism. We hypothesize that the genome-wide data obtained in the resting somatic cells reflect a "maintenance stage" in the gene activation process (Figure 5c). In our novel model, we propose that HATs could have a dual mode of action by distinguishing "initiation" and "maintenance" stages during the transcription activation process. The "initiation" stage would reflect the initial steps in the switch from an inactive to active transcription state, while the "maintenance" stage would represent the stabilisation of an active transcription stage over the time. In the initiation stage, the transcriptional activation of a given gene would be highly dependent on the specific recruitment of one given HAT (Figure 5c, left). Following this "initiation" stage, through a sequential process, this initial activation would lead to the consequent recruitment or binding of multiple HATs, which may recognize the initially open and acetylated environment in a less specific manner. This hypothesis is in good agreement with the findings that many HAT-containing complexes contain subunits with bromodomains that are thought to bind to acetylated histone tails. The binding of several HATs to the initially acetylated locus would thus serve to maintain the acetylation level and by consequence the activation state of a given locus (Figure 5c, right).

Our model would thus explain why in dynamic developmental processes HATs show high functional specificity (genetic data) that may not be the case in resting differentiated somatic cells. During cell differentiation, the high complexity level of transcriptional regulatory networks is a prerequisite for the orchestration of the time-controlled initiation of the proper transcriptional programs. However, in resting somatic tissues, where the number of newly initiated transcription processes is likely to be lower than during cell commitment, the large panel of activators would redundantly maintain activation states. This could explain why high redundancy in the recruitment of HATs is observed in resting cells, while their functional specificity is needed to achieve proper embryo development.

The above-presented data [12] is focused on catalytic subunits of HAT complexes. However, as discussed previously, a given HAT enzyme is (with the notable exception of CBP/p300) often embedded in more than one large multisubunit complex. Therefore, although the distinct HAT enzymes appear to be corecruited at many loci, they can represent the occurrence of different HAT complexes. Indeed, two recent studies analysed the differences of the recruitment between the two MOF-containing complexes (called NLS and MLS) at the genome-wide scale in *Drosophila *[24,25]. The studies, by targeting NLS- or MLS-specific subunits, show a high specificity in the recruitment of these complexes. Thus, by studying individually the well-defined HAT complexes, the resolution of the analysis will increase, leading to a more precise separation of the observed overlap in the recruitment of the HATs. Nevertheless, the existence of specific loci, where a single HAT is recruited should not be excluded. However, according to the analysis of the data from the Zhao lab [12], these events appear to be the exception rather than the rule.

The very recent improvement of the genome-wide mapping technologies and the development of novel ChIP-grade antibodies against HATs and complex-specific subunits will allow researchers to verify these genome-wide observations and provide new insights into the functional roles of HATs. For example, the time course analysis of different HAT recruitments upon dynamic activation of a particular gene networks, for example, *in vitro *stem cell differentiation systems [26], should allow researchers to test the proposed model.

## Conclusions

HAT-containing complexes are key components of chromatin-mediated transcriptional regulatory networks. Proper understanding and modelling of their mode of action and function within these networks is a prerequisite for accurate prediction of the behaviour of transcriptional systems. Recent technology breakthrough in the postgenomic area allowed new insights into the function of HATs, but were often interpreted with the liability of ignoring previously available biochemical and genetic data, leading to oversimplified models. Here we propose novel considerations that could reconcile the different lines of evidence in a unified model that describes the mode of action of HATs more precisely. Our current model proposes that HATs are differently required, depending on the stage of gene activation, with a high functional specificity in the early gene activation stage and with a less-specific functionality in the later maintenance stage.

## Competing interests

The authors declare that they have no competing interests.

## Authors' contributions

ARK, KA and LT contributed to the study design. KA and ARK performed the bioinformatics analyses. KA, ARK, DD, JT, OP and LT wrote the manuscript. All authors read and approved the final manuscript.
